# Main crustal seismic sources in El Salvador

**DOI:** 10.1016/j.dib.2018.08.147

**Published:** 2018-08-31

**Authors:** Jorge Alonso-Henar, María Belén Benito, Alejandra Staller, José Antonio Álvarez-Gómez, José J. Martínez-Díaz, Carolina Canora

**Affiliations:** a Universidad Complutense de Madrid, Department of Geodynamics, Madrid, Spain; b Universidad Politécnica de Madrid, Department of Topography Engineering and Mapping, Spain; c Instituto de Geociencias IGEO (UCM,CSIC), Madrid, Spain; d Universidad Autónoma de Madrid, Department of Geology and Geochemistry, Spain

## Abstract

We present a map and a data set containing information about intra-plate seismic sources in El Salvador. These are the results of the field campaigns and data analysis carried out by the research group of Planetary Geodinamics, Active Tectonics and Related Risks from Complutense University of Madrid during the last 12 years. We include two maps, the first map contains 1405 fault traces with evidences of Quaternary activity derived form morphometric, paleoseismological and geomorphological analysis together with field data mapping carried in El Salvador. The second map is a synthesis of the 29 intra-plate seismic sources selected from the quaternary faults map. The geometry of these sources was simplified and we also include a table where some available data of the proposed sources are included, such as their name, orientation, length and slip-rate. For further interpretation and discussion of these sources see (Alonso-Henar et al., 2018) [1, doi.org/10.1016/j.enggeo.2018.06.015].

**Specifications table**

**Table:** 

Subject area	*Geology, Physics*
More specific subject area	*Seismic Hazard*
Type of data	*Table and figures*
How data was acquired	*Field studies, Teledetection, Geodesy*
Data format	*Raw, Filtered, Analyzed*
Experimental factors	
Experimental features	*Geological mapping and paleoseismology*
Data source location	*El Salvador*
Data accessibility	

**Value of the data**

• These data have a direct influence on the El Salvador Seismic Hazard Assessment.
• We strongly recommend the use of these sources in future seismic hazard studies in El Salvador.
• We emphasize the necessarily collaborations with geo-researchers in order to enhance the knowledge of seismic sources in El Salvador.
• These data are the seed of the Quaternary Active Fault Database of El Salvador (QAFES), in preparation, whose methodological proposal was presented in [2]. Further studies are necessary to complete the map of seismic sources, specially in the northern part of the country and to obtain additional activity parameters of the faults within the Salvadoran Volcanic Arc.

## 1 Data

The data that exposed are main and minor fault traces and available features of the El Salvador Fault Zone, an active strike-slip fault zone that crosses the country from east to west [3]. This fault zone is developed within the Central America Volcanic Arc. It is composed of main strike slip faults trending N 90°-110°E and secondary normal faults trending between N 120°-170°E. The seismic potential of this fault zone has been demonstrated during the 13th February 2001 Mw 6.6 destructive earthquake (but higher magnitudes are expected in future earthquakes, see [1] associated research article).

## 2 Experimental design, materials and methods

**Fig. 1:**
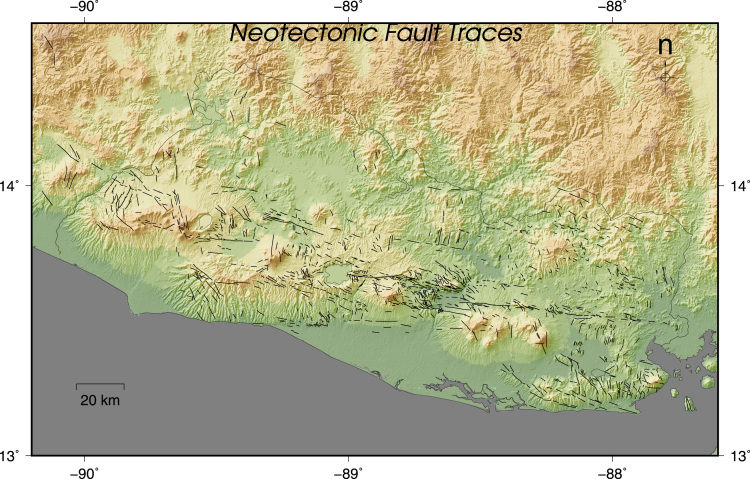
Qauternary Fault Traces used for the mapping of the seismic sources in Fig. 2.

The faults presented here are divided in two maps and one table. The first map contains the fault traces of faults with evidences of Quaternary activity mapped from morphotectonic, paleseismological and field studies and the compilation of data published in several research articles and maps (Fig. 1) [2], [3], [4], [5], [6], [7], [8], [9], [10], [11]. The faults of the first map do not necessary accommodate elastic deformation, these traces indicate strictly the places where morphological features (mainly fault scarps or offsets) and/or stratigraphical features (displacement of young rock formations) were identified. There is no kind of interpolation between fault traces and there is no information about the activity of most of them.

**Table 1:** Seismic sources of El Salvador.

Fault ID	NAME	SECTOR	STRIKE	DIP	*LENGHT*	SR (mm/yr)
1	Intipuca	Jucuarán-Intipucá	288	70	25,652	
2	Olomega	Jucuarán-Intipucá	318	70	12,627	
3	La Quesadilla	Jucuarán-Intipucá	320	70	10,987	
4	El Zapote	Jucuarán-Intipucá	185	70	11,177	
5	Conchagua	Jucuarán-Intipucá	189	70	13,490	
6	Chilanguera	Jucuarán-Intipucá	300	70	5832	
7	Rio Grande	Jucuarán-Intipucá	309	70	19,108	
8	El Espino	Jucuarán-Intipucá	288	70	11,016	
9	Chirilagua	Jucuarán-Intipucá	167	70	14,460	
10	Guachipilin	Lempa	116	70	19,764	
11	Tecomatal	Lempa	132	70	15,369	
12	El Pulguero	Lempa	106	70	23,096	
13	Guaycume	Western Segment	108	70	23,665	9 ± 3 (1)
14	Comecayo	Western Segment	96	70	19,670	
15	Santa Ana W	Western Segment	344	60	14638	
16	Santa Ana E	Western Segment	163	60	13,443	
17	Apaneca	Western Segment	311	90	14,384	
18	Teotepeque	Western Segment	275	90	6761	
19	El Zacamil	Western Segment	359	90	12,066	
20	Sesuntepeque E	North Lempa	193	60	14,820	
21	Sesuntepeque W	North Lempa	177	60	13,121	
22	Victoria	North Lempa	193	90	12,294	
23	San Vicente	San Vicente	88	70	18,774	7 ± 1 [12], 4 [6]
24	El Triunfo	Berlin Segment	94	70	28,251	7.5 ± 3.5 [12], 11 [5], 4.8 [9]
25	Coatepeque	Western Segment	94	90	17,891	
26	San Miguel	San Miguel	94	90	34,152	
27	Lempa Sur	Berlin Segment	277	90	10,411	
28	Berlin Fault	Berlin Segment	278	90	12,391	
29	El Caracol	Western Segment	271	90	12,595	

**Fig. 2:**
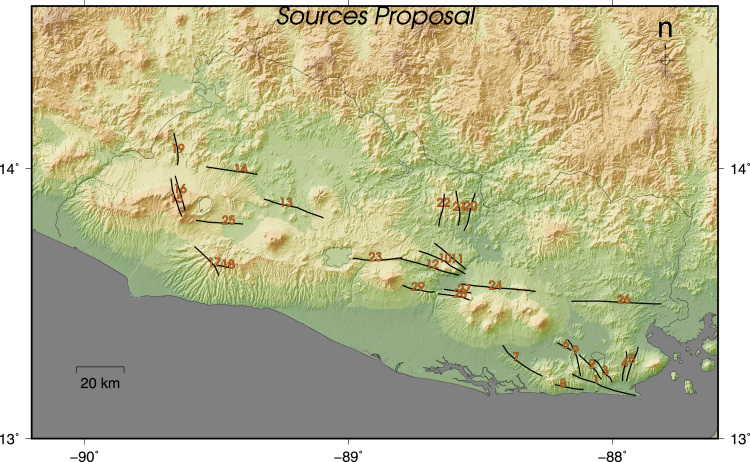
Seismic Sources Traces of El Salvador. Numbers are the Fault ID in Table 1.

The second map is a seismic source proposal inferred from the first map, where appear the fault traces of the main faults that may accommodate elastic energy and/or have evidences of Quaternary activity, identified by offsets, morphometry, GPS studies or paleoseismological trenching (Fig. 2) [6], [7], [9], [12]. In the Table there are summarized the main features of this faults when they are available.

## References

[1] Alonso-Henar J., Benito B., Staller A., Álvarez-Gómez J.A., Martínez-Díaz J.J., Canora C. (2018). Large-magnitude crustal seismic sources in El Salvador and deterministic hazard scenarios. Eng. Geol..

[2] Martínez-Díaz J.J., Canora C., Insua-Arevalo J.M., Álvarez-Gómez J.A., Alonso-Henar J., Staller A., Villamor P., Díaz M., Hernández D., Hernández W. (2017). Propuesta metodológica para la base de datos de fallas activas de El Salvador (Methodological proposal for the QAFES: Quaternary Active Faults of El Salvador).

[3] Martínez-Díaz J.J., Álvarez-Gómez J.A., Benito B., Hernández D. (2004). Triggering of destructive earthquakes in El Salvador. Geology.

[4] Bosse H.R., Lorenz W., Merino A., Mihm A., Rode K., Schmidt-Thomé M., Wiesemann G., Weber H.S. (1978). Geological Map of El Salvador Republic: Hannover Germany.

[5] Corti G., Carminati E., Mazzarini F., Garcia M.O. (2005). Active strike-slip faulting in El Salvador, Central America. Geology.

[6] Canora C., Martínez-Díaz J.J., Villamor P., Berryman K., Álvarez-Gómez J.A., Pullinger C., Capote R. (2010). Geological and seismological analysis of the 13 February 2001 Mw 6.6 El Salvador earthquake: evidence for surface rupture and implications for seismic hazard. Bull. Seismol. Soc. Am..

[7] Canora C., Villamor P., Martínez-Díaz J.J., Berryman K., Álvarez-Gómez J.A., Capote R., Hernández W. (2012). Paleoseismic analysis of the San Vicente segment of the El Salvador Fault Zone, El Salvador, Central America. Geol. Acta.

[8] Canora C., Martínez-Díaz J.J., Insua-Arévalo J.M., Álvarez-Gómez J.A., Villamor P., Alonso-Henar J., Capote-Villar R. (2014). The 1719 El Salvador Earthquake: an M > 7.0-7:0 Event in the Central American volcanic arc?. Seism. Res. Lett..

[9] Alonso-Henar J., Álvarez-Gómez J.A., Martínez-Díaz J.J. (2014). Constraints for the recent tectonics of the El Salvado Fault Zone, Central America Volcanic Arc, from morphotectonic analysis. Tectonophysics.

[10] Alonso-Henar J., Schreurs G., Martinez-Díaz J.J., Álvarez-Gómez J.A., Villamor P. (2015). Neotectonic development of the El Salvador Fault Zone and implications for deformation in the Central America Volcanic Arc: insights from 4-D analog modeling experiments. Tectonics.

[11] Alonso-Henar J., Álvarez-Gómez J.A., Martínez-Díaz J.J. (2017). Neogene-quaternary evolution from transpressional to transtensional tectonics in Northern Central America controlled by cocos: caribbean subduction coupling change. J. Iber. Geol..

[12] Staller A., Martínez-Díaz J.J., Benito B., Alonso-Henar J., Hernández D., Hernández-Rey R., Díaz M. (2016). Present-day crustal deformation along the El Salvador Fault Zone from ZFESNet GPS network. Tectonophysics.

[13] Wessel P., Smith W.H.F., Scharroo R., Luis J.F., Wobbe F. (2013). Generic mapping tools: improved version released. Eos Trans. AGU.

